# Aldehyde Dehydrogenase, a Therapeutic Target in Chordoma: Analysis in 3D Cellular Models

**DOI:** 10.3390/cells10020399

**Published:** 2021-02-15

**Authors:** Marie-Anaïs Locquet, Anne-Lise Dechaume, Paul Berchard, Lhorra Abbes, Daniel Pissaloux, Franck Tirode, Inès Ramos, Julie Bedoucha, Julie Valantin, Marie Karanian, Raul Perret, Olivier Gille, Jean-Yves Blay, Aurélie Dutour

**Affiliations:** 1 Team Cell Death and Pediatric Cancer, Cancer Initiation and Tumor Cell Identity Department, INSERM1052, CNRS5286, Cancer Research Center of Lyon, F-69008 Lyon, France; Marieanais.locquet@lyon.unicancer.fr (M.-A.L.); Annelise.dechaume@lyon.unicancer.fr (A.-L.D.); Paul.berchard@lyon.unicancer.fr (P.B.); Lhorra.abbes@lyon.unicancer.fr (L.A.); ines.laurentramos@gmail.com (I.R.); julie.bedoucha@gmail.com (J.B.); Jean-yves.blay@lyon.unicancer.fr (J.-Y.B.); 2 Department of Biopathology, Centre Leon Berard, F-69008 Lyon, France; Daniel.pissaloux@lyon.unicancer.fr; 3 Team Genetics, Epigenetics and Biology of Sarcomas, Univ Lyon, Université Claude Bernard Lyon 1, INSERM1052, CNRS5286, Cancer Research Center of Lyon, Centre Leon Berard, F-69008 Lyon, France; Franck.tirode@lyon.unicancer.fr (F.T.); Marie.karanian@lyon.unicancer.fr (M.K.); 4 Research Pathology Platform, Department of Translational Research and Innovation, Centre Leon Berard, F-69008 Lyon, France; Julie.valantin@lyon.unicancer.fr; 5 Fondation Synergie Lyon Cancer, F-69008 Lyon, France; 6 Department of Biopathology, Institut Bergonié, F-33000 Bordeaux, France; r-perret@bordeaux.unicancer.fr; 7 Orthopedic Spinal Surgery Unit 1, Bordeaux University Hospital, F-33000 Bordeaux, France; Olivier.gilles@chubordeaux.fr; 8 Medical Oncology Department, Centre Leon Berard, F-69008 Lyon, France

**Keywords:** chordoma, 3D models, hypoxia, radiotherapy, aldehyde dehydrogenase, radioresistance, combination therapy

## Abstract

Chordomas are rare, slow-growing tumors of the axial skeleton. These tumors are locally aggressive and refractory to conventional therapies. Radical surgery and radiation remain the first-line treatments. Despite these aggressive treatments, chordomas often recur and second-line treatment options are limited. The mechanisms underlying chordoma radioresistance remain unknown, although several radioresistant cancer cells have been shown to respond favorably to aldehyde dehydrogenase (ALDH) inhibition. The study of chordoma has been delayed by small patient cohorts and few available models due to the scarcity of these tumors. We thus created cellular 3D models of chordoma by using low-adherence culture systems. Then, we evaluated their radiosensitivity using colony-forming and spheroid size assays. Finally, we determined whether pharmacologically inhibiting ALDH increased their radiosensitivity. We found that 3D cellular models of chordoma (derived from primary, relapse, and metastatic tumors) reproduce the histological and gene expression features of the disease. The metastatic, relapse, and primary spheroids displayed high, medium, and low radioresistance, respectively. Moreover, inhibiting ALDH decreased the radioresistance in all three models.

## 1. Introduction

Chordomas are rare bone tumors of the axial skeleton that localize preferentially in the cranial and the sacral areas. With an incidence of 700 new cases per year, chordomas account for 1–4% of bone tumors and 20% of spinal tumors [1]. These tumors, thought to arise from embryonic remnants of the notochord, can affect people at any time through life even if the median age at diagnosis is between 50 and 60 years [2,3]. Conventional chordomas possess some peculiar characteristics. They are slow growing tumors and are locally aggressive, invading surrounding structures including the bone and often compressing important structures such as cranial nerves or the spinal cord [4]. They are almost avascular, leading to strong hypoxia, and are composed of large, vacuolated, physaliphorous cells surrounded by an abundant mucoid extracellular matrix [5].

As in the vast majority of bone sarcoma, chordoma patient management consists of surgical resection of the tumor [6]. The goal of resection is to obtain adequate margins, and the quality of surgery is the most important prognostic factor [4]. However, resection with adequate margins is achieved in roughly 50% of cases, mainly due to the location of the tumor in the vicinity of important structures [4]. In the case of inadequate margins, neoadjuvant therapy is combined with surgery. Among all neoadjuvant therapies, high-dose conventional radiotherapy remains the only option, with doses superior to 70 Gy [7,8]. Despite its low accessibility, proton therapy can be useful because it allows the delivery of a more important dose to a tumor without exposing surrounding tissues to excessive toxicity [9]. Despite this aggressive treatment, the 5-year relapse-free survival rate is only 50% [8]. Treatment options are limited at relapse due to the invasiveness and toxicity of the first-line treatment. Hence, there is a need for therapies addressing chordoma specificities. To achieve this, a better understanding of chordoma biology and of the mechanisms underlying chordoma radioresistance is of utmost importance. Decreasing the radioresistance of these tumors could decrease the toxicity triggered by radiations, could improve patient care, and could improve tumor control. The scarcity of these tumors makes large-scale molecular studies challenging, and only a few molecular alterations have so far been described [10]. Although the causes of radioresistance in chordoma are still largely unknown, several factors may be involved. Indeed, hallmarks of these tumors are their slow growth and their low number of mitotic cells, which are the principal targets of radiotherapy [11]. Moreover, the low level of vascularization leads to strong hypoxia, which represents a major predictive factor of unfavorable tumor response to radiotherapy [12,13].

To improve our understanding of the mechanisms underlying chordoma radioresistance, we need to reconstitute the peculiar characteristics of these tumors. Because of their scarcity, only a few models of these tumors are available: 20 cell lines and 11 patient-derived xenograft models. Moreover, cells grown in 2D do not reflect some of the major characteristics of chordoma such as hypoxia, extracellular matrix production, and disease progression [14]. To address these issues, we hypothesized that creating cellular 3D models of chordoma could improve our understanding of the mechanisms of radioresistance. Thus, the aims of our study were (i) to create cellular 3D models of chordoma that reproduce their histological and morphological features, (ii) to evaluate their resistance to radiations, and (iii) to determine whether a radiosensitizing approach could be applicable. In this regard, we tested the effectiveness of targeting aldehyde dehydrogenases (ALDHs) 1 and 3. Indeed, ALDHs are a family of enzymes involved in cell detoxification [15]. They oxidize a wide range of endogenous and exogenous aldehydes and protect living organisms from oxidative stress [6]. A high ALDH activity has already been correlated with resistance to treatment in multiple solid tumors. For instance, ALDH-positive breast cancer cells are radioresistant in vitro [16,17]. ALDH1-positive cells exhibit a radioresistant phenotype enhanced with hypoxia in cervical cancer [18]. ALDH activity promotes radioresistance in prostate cancer progenitor cells [19] and is indicative of head and neck squamous cell carcinoma (HNSCC) radioresistant cells [20]. This is also the case for bone sarcoma, in which a high ALDH activity is associated with chemotherapy-resistant Ewing sarcoma cells [21]. A population of cells with a high ALDH activity is known to be present in both chordoma patients [22] and cell lines U-CH1 [23], U-CH2, UM-Chor1 [24], and JHC7 [25]. Moreover, the inhibition of ALDH decreases the radioresistance of breast, prostate, and cervical cancer cells [26,27,28]. Collectively, these findings suggest a central role for ALDH in cellular resistance to radiations. However, the involvement of ALDH in radioresistance has never been studied in chordoma.

Hence, we created cellular 3D models of chordoma by using ultra-low adherence culture systems. We evaluated their radiosensitivity and determined whether pharmacologically inhibiting ALDH1/3 decreased the radioresistance of our models.

## 2. Materials and Methods

### 2.1. Cell Lines

#### 2.1.1. Cultures

Conventional chordoma cell lines U-CH12, U-CH1, and CH22 established from primary, relapse, and metastatic chordoma, respectively, were kindly provided by the Chordoma Foundation (Durham, NC, USA). The U-CH1 and U-CH12 cell lines were maintained in 2D cultures on rat tail collagen I-coated flasks (A1048301, Thermofisher, Illkirch, France) in Iscove Modified Dulbecco Media (IMDM):Roswell Park Memorial Medium (RPMI) 4:1 medium (Life technologies, Carlsbad, CA, USA) supplemented with insulin, transferrin, and selenium 1% (ITS, Life technologies) for U-CH12. The CH22 cell line was maintained on non-coated flasks in RPMI medium. All media were supplemented with 10% fetal bovine serum (FBS) and 1% penicillin streptomycin (PS).

#### 2.1.2. Cell Culture Conditions and Treatments

Chordoma spheroids were generated by seeding 2500 cells from either cell lines in 96 ultra-low attachment (ULA) plates (Corning^®^ Costar^®^, Brumath, France) in the same media as used for 2D cultures. Once seeded, spheroid formation was examined twice a week under an optical inverted microscope (Zeiss, Göttingen, Germany). Spheroids were allowed to form for 7 days before being treated either with radiation or an ALDH inhibitor. A 2 Gy radiation dose was applied to spheroids (dose rate of 6 Gy min^−1^) with a 6 MeV X-ray clinical irradiator (SL 15 Phillips). The irreversible inhibitor of ALDH1 and ALDH3, DIMATE (Advanced BioDeisgn, Saint Priest, France), was added to spheroids for 24 to 72 h (concentrations range: 0.1 to 25 μM).

### 2.2. Proliferation Assay

Spheroid proliferation was assessed at days 4, 7, 10, 15, and 21 by CellTiter-Glo^®^ luminescent cell viability assay (CTG) (Promega, Charbonnières-les-Bains, France). Each spheroid was washed with PBS1X and incubated with 60 µL of CTG in a medium devoid of serum (ratio 1:1). Luminescence was detected after 25 min of incubation on a TECAN Infinite 500 reader (TECAN, Männendorf, Switzerland) for 500 ms/well. After 48 h of treatment, spheroid proliferation in response to increasing concentrations of DIMATE (0.1 to 25 μM) was measured by Cell Titer Glo to determine DIMATE IC50.

### 2.3. Real-Time Monitoring

Spheroid proliferation was also determined using real-time monitoring with an IncuCyte Live Cell Analysis System (Incucyte ZOOM, Essen Bioscience, West Wickham Kent BR4 OPH, UK). Spheroid diameters were measured using the IncuCyte software. Spheroid cell death induced by radiation and/or DIMATE treatment was monitored in real-time using the IncuCyte. Irradiated and/or DIMATE-treated spheroids were incubated with 50 μL of Cytotox Green reagent (4633, Essen Bioscience) in phenol red-free medium according to the manufacturer’s instructions and placed under the 20× objective lens of the IncuCyte Live Cell Analysis System over a period of 48 h. The evolution of Cytotox Green fluorescence intensity was then analyzed with the IncuCyte software.

### 2.4. Immunostaining

Spheroids were harvested at day 7, 10, 15, and 21 after seeding; fixed in eosin 1:100 and PFA 4% (2 h, 4 °C); embedded in paraffin before cutting into 5 µm sections; and stained. HPS and Ki67 staining were performed on an automated Ventana Discovery XT staining system (Ventana Medical Systems, Innovation PARk Drive, Roche, Tucson, AZ 85,755, USA). The Ki67 index was assessed semi-quantitatively. For hypoxia staining, the sections were incubated with pimonidazole (100 µg/mL for 1 h at room temperature (RT) (Hypoxyprobe, MA, USA) before fixation.

After the deparaffination, rehydration, and antigen retrieval steps (citrate buffer, pH 6.0, 95 °C, 15 min or Tris EDTA, 95 °C, 15 min), the slides were incubated overnight (4 °C) with the following primary antibodies: anti-brachyury(1:500), anti CD24 (1:100), 4-hydroxynonenal (Abcam, Cambridge, UK), anti-cytokeratin AE1/AE3 (1:100), anti EMA (1:250) (Dako, Agilent, Santa Clara, CA, USA), anti γH2AX (1:500) (Cell signaling technology, Leyde, The Netherland), anti HIF-1α (1:100), anti-ALDH3A2 (1:100) (Proteintech, Manchester, UK), or anti-pimonidazole (Hypoxyprobe). Endogenous peroxidases were inactivated by incubating the slides in oxygen peroxide (0.3%, 15 min, RT). The primary antibodies were detected with biotinylated goat anti-rabbit or goat anti-mouse secondary antibody (VectorLab, Burlingame, United States, 1:100, 1 h, RT) followed by an avidin-biotin complex and DAB peroxidase (SK-4100, Vector Lab; dilution 1:300, 30 min, RT). The sections were counterstained in hematoxylin (Vector Lab, H3401) and mounted in Vectamount medium (H-5000). For aldehyde staining, the sections were incubated with secondary antibody (goat anti-mouse Alexa fluor 488, Abcam, 150113) and DAPI (1:1000, Abcam, 228549) for 1 h at RT. The sections were then mounted on slides using Fluoromount (Sigma, Merck, Darmstadt, Germany). For extracellular matrix staining, the slides were incubated with alcian blue (Sigma, B8438) and sirius red (Sigma, 2610) for 10 min at RT. The sections were washed and counterstained with fast red or hematoxylin, respectively. All slides were examined under a Zeiss Axioimager M2 microscope (SIP 60549, Zeiss, Göttingen, Germany), and quantifications were made using the ImageJ software.

### 2.5. Electron Microscopy

Spheroids were fixed in buffered aldehyde (4% formaldehyde, 2% glutaraldehyde, 1 mM MgCl_2_, and 1 mM CaCl_2_ in 100 mM Ca-cacodylate, pH 7.2), post-fixed in aqueous 1% osmium tetroxide, and then dehydrated in graded steps of ethanol. The spheroids were impregnated in Epon A (50%), Epon B (50%), and DMP30 (1.7%) and were included for polymerization (60 °C, 72 h). The spheroids were cut in ultrathin 70 nm sections before being contrast-stained with lead-citrate and uranyl acetate and imaged with a Jeol 1400JEM (Tokyo, Japan) transmission electron microscope at the CIQLE (Centre d’Imagerie Quantitative Lyon Est).

### 2.6. Self-Renewal

Self-renewal of the chordoma spheroids was assessed by colony formation assays and spheroid size assays. After irradiation and/or DIMATE treatment, the spheroids were maintained 24 h in culture before being dissociated using the TryPLE reagent (5 min, 37 °C, Thermofisher, Illkirch, France) followed by gentle pipetting to obtain single cell suspensions.

Two thousand or five thousand cells were seeded onto 6-well plates and were allowed to form colonies for 5 doubling times (2 weeks for CH22, 5 weeks for U-CH12, and 7 weeks for U-CH1). The colonies were counterstained with a solution of ethanol 75% crystal violet (Sigma, V5265) and counted using the ImageJ software. To perform a spheroid size assay, 200 single cells were seeded in ULA 96-well plates and were allowed to form spheres for 15 days. All spheres (>50 μm) in a well were manually imaged, counted, and measured (diameter) using a Zeiss Axio Observer Z1 inverted microscope (Zeiss, Göttingen, Germany).

### 2.7. Aldefluor Assay

ALDH enzymatic activity was measured using the Aldefluor kit (Stem Cell Technologies). The cells suspended in Aldefluor assay buffer were incubated with an ALDH enzyme substrate for 40 min at 37 °C. As a control for baseline fluorescence, the cells were also treated with ALDH inhibitor diethylaminobenzaldehyde (DEAB). The cells were washed and stained with viability dye (Miltenyi Biotec, Paris, France) at 1:1000. Fluorescence was detected using a BD Bioscience LSR Fortessa flow cytometer and analyzed using the FACSDiva software (BD Biosciences, San Jose, CA, USA).

### 2.8. RNA Sequencing

RNA sequencing was performed for both spheroids after 7 days of culture and patient samples. Total RNA was extracted from macro-dissected formalin-fixed paraffin-embedded tumor sections using the FormaPure RNA kit (Beckman Coulter #C19158, Brea, CA, USA). The RNase-free DNase set (Qiagen #AM2222, Courtaboeuf, France) was used to remove DNA. RNA quantification was assessed using NanoDrop 2000 (Thermo Fisher Scientific, Waltham, MA, USA) measurement and RNA quality using theDV200 value (the proportion of RNA fragments larger than 200 nt) assessed by a TapeStation with Hs RNA ScreenTape (Agilent, Santa Clara, CA, USA). Samples with sufficient RNA quantity (>0.5 μg) and quality (DV200 > 30%) were further analyzed by RNA sequencing. One-hundred nanograms of total RNA were used to prepare libraries with the TruSeq RNA Exome (Illumina #20020183, San Diego, CA, USA). Twelve libraries were pooled at a concentration of 4 nM each, together with 1% PhiX. Sequencing was performed (paired end, 2 × 75 cycles) using NextSeq 500/550 High Output V2 kit on a NextSeq 500 machine (Illumina).

Alignments were performed using STAR on the GRCh38 version of the human reference genome. The number of duplicate reads were assessed using PICARD tools. No sample was discarded from the analysis (number of unique reads above 10 million). The expression values were extracted using Kallisto version 0.42.5 tool17 with GENECODE release 23-genome annotation based on the GRCh38 genome reference. The Kallisto TPM expression values were transformed in log2(TPM+2), and all samples were normalized together using the quantile method from the R limma package within R (version 3.1.1) environment. tSNE analysis was performed to visualize the distance between chordoma spheroids, patient samples, and a series of 1450 sarcoma samples with at least 144 molecular subtypes, as previously published [29]. A classic differential gene expression analysis was conducted between chordoma samples and spheroids.

### 2.9. Patients Samples and Ethics

The study was approved by the Clinical Research Ethics Committee of the Centre Leon Berard, ethical approval number 2018-014, approval date 3 October 2018. A total of 13 patients with conventional chordoma were included in the study. The clinical informations are presented in Table 1.

### 2.10. Statistical Analysis

GraphPad Prism (GraphPad Software, Inc., La Jolla, CA, USA) v8.0 was used for the graphic representation and statistical analysis of the data. The comparison between groups in all experiments was determined using analysis of variance in GraphPad v8.0. Tukey’s test or Sidak’s test were used in the post analysis for one-sided and two-sided statistical analyses, respectively. The differences were considered significant if the *p*-value was ≤0.05.

## 3. Results

### 3.1. Chordoma Spheroids Recapitulate the Main Histological and Morphological Features of the Disease

Because the few available cellular models of chordoma do not fully recapitulate the structures of these tumors, we generated 3D cellular models of chordoma originating from three cell lines representative of the three different stages of the disease: (i) primary tumor U-CH12, (ii) locally relapsed tumor U-CH1, and (iii) metastatic tumor CH22. We first investigated whether these spheroids faithfully reproduced the most prominent histological and morphological features of the disease.

**Figure 1:**
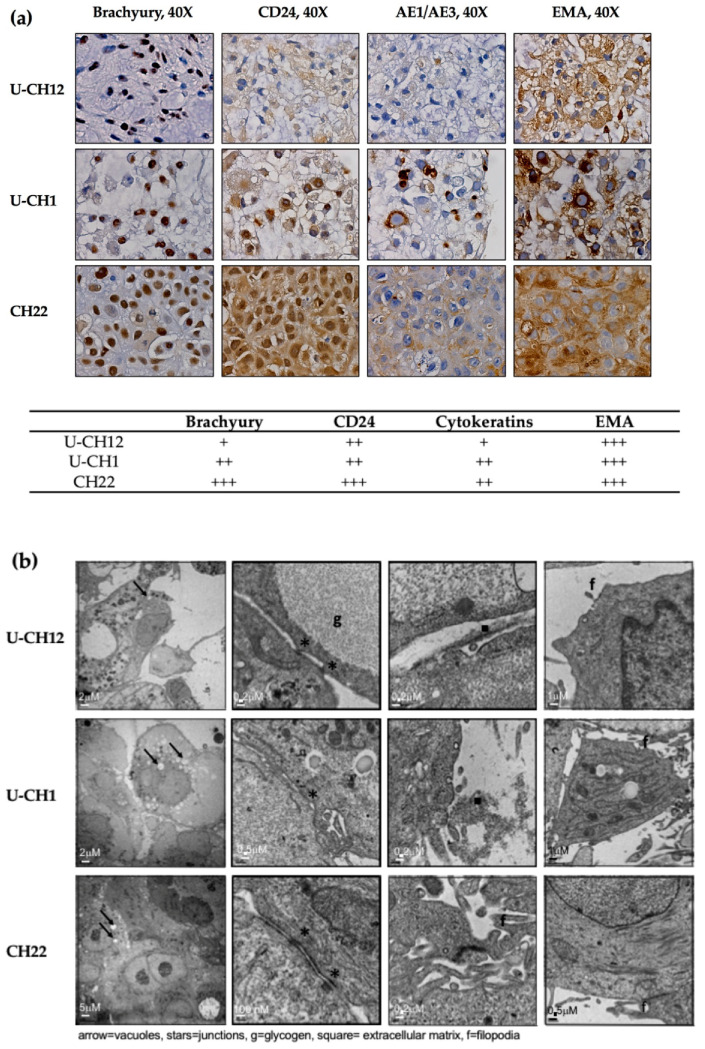
Chordoma spheroids recapitulate the main histological and morphological features of the disease: at 7 days of culture, (**a**) chordoma markers brachyury, CD24, cytokeratins AE1/AE3, and EMA (40× objective) and their quantification were immunostained and (**b**) spheroid morphology was imaged by an electron microscopy. The arrows represent vacuoles, the stars represent junctions, the squares represent the extracellular matrix, f is the filopodia, and g is the glycogen granules.

At the histological level, all three spheroid models expressed the chordomas markers Brachyury, CD24, cytokeratins, and EMA (Figure 1a). While EMA was strongly expressed by the three chordoma spheroid models, Brachyury and CD24 were highly expressed by the CH22 and U-CH1 spheroids and the U-CH12 spheroids displayed a low expression level. Finally, the CH22 spheroids alone presented a high expression of cytokeratins (Figure 1a).

Morphologically, consistent with the physaliphorous phenotype of chordoma, spheroid cytoplasm included large vacuoles. Their cytoplasm also held glycogen granules, small aggregates of tonofilaments, and rough endoplasmic reticulum around the mitochondria (Figure 1b). All spheroids presented prominent surface filopodia. At the cellular level, CH22 spheroids were characterized by very cohesive cells CH22, joined by tight junctions, whereas U-CH1 and U-CH12 spheroids were less cohesive, only displaying zones of contact between cells (Figure 1b).

Hence, the 3D spheroid models generated recapitulated the main histological and morphological features of chordoma.

Finally, to confirm the validity of our models, we performed RNA sequencing on a cohort of 13 patients and on our spheroids and compared their repertoire of expression to 1450 sarcoma samples, forming at least 144 molecular subtypes. Visualization by tSNE clearly showed that patient samples clustered close to our spheroid samples (Figure S1A). Moreover, a differential gene expression analysis was conducted, highlighting the close relationship between chordoma samples and spheroids (Figure S1B and Tables S1 and S2). These results strongly reinforce the validity of our models.

### 3.2. Chordoma Spheroids Recapitulate the Radioresistant Environment of the Disease

Other chordoma characteristics are the presence of a mucoid extracellular matrix, a strong hypoxia, and a slow progression, at least in their localized form. We then investigated whether our chordoma spheroids present these features.

**Figure 2:**
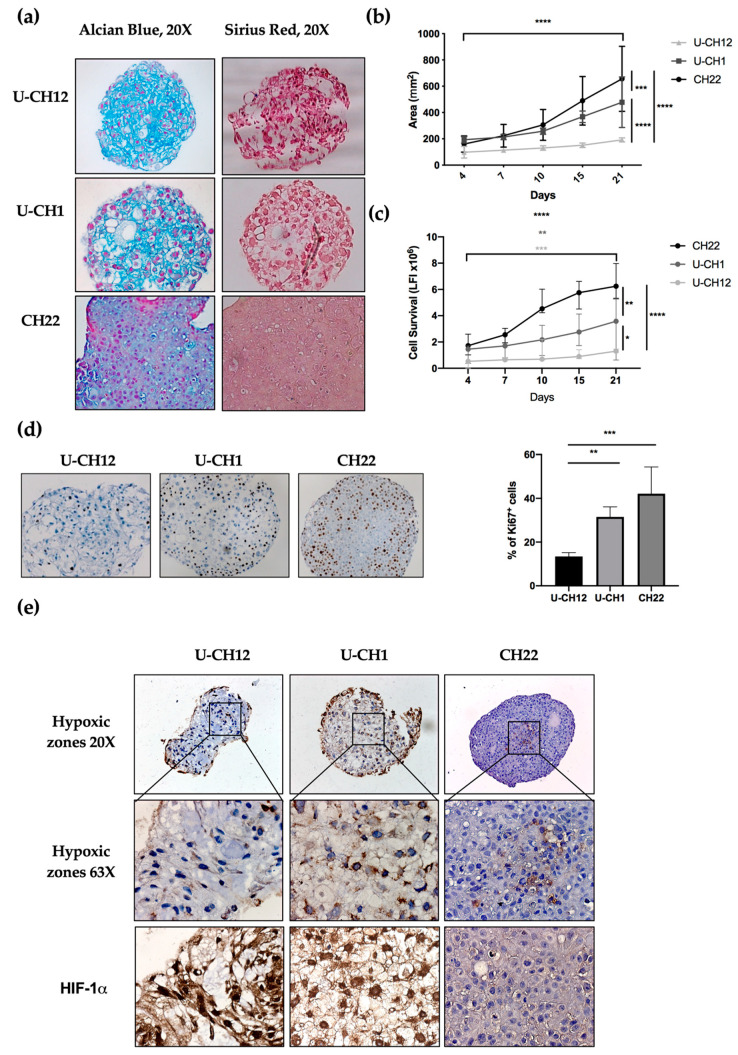
Radioresistant chordoma spheroid environment: at 7 days of culture, (**a**) extracellular matrix components were immunostained, including proteoglycans with alcian blue and collagen with sirius red (20× objective). (**b**) The graph represents the median and confidence interval at 95% of the measure of the area of spheroids using live imaging from day 0 to day 21 after seeding for each spheroid. (**c**) The graph represents the median and confidence interval at 95% of the luminescence fluorescence intensity (LFI) correlated with cell survival from day 0 to day 21 after seeding for each spheroid. (**d**) The number of proliferative Ki67+ cells was evaluated, and the median and confidence intervals at 95% for each spheroid were quantified. (**e**) The hypoxic zones within spheroids were mapped by pimonidazole and HIF-1α staining. Each experiment was conducted in triplicate and repeated three times for spheroid proliferation assessment or twice for extracellular matrix and hypoxia-relative staining. For a comparison of the areas and cell survival, statistical analysis included 2-way ANOVA with time comparison, presented at the bottom of each graph, and cell line comparisons, presented on the right-hand side of the graphs. The comparison of the number of Ki67+ cells between spheroids was determined using a one-way ANOVA analysis. Significant *p*-values are indicated as follows: *p* < 0.05 *, *p* < 0.01 **, *p* < 0.001 ***, and *p* < 0.0001 ****.

The presence and composition of ECM in chordoma spheroids was analyzed by sirius red and alcian blue staining, enabling us to visualize collagen and proteoglycans, two major ECM components. Two spheroids U-CH12 and U-CH1 produced their own ECM (Figure 2a). U-CH12 ECM was rich in collagen fibers, as shown by a more intense sirius red staining, while both U-CH1 and U-CH12 spheroid ECMs contained abundant proteoglycans, as evidenced by alcian blue staining (Figure 2a).

Next, we evaluated spheroid growth by measuring their area over a period of 21 days through live imaging (Figure 2b). CH22 spheroids reached a size of 180 mm^2^ on day 4 to 740 mm^2^ on day 21 (*p* < 0.0001). U-CH1 spheroids were less proliferative (*p* < 0.001) and grew from 190 mm^2^ on day 4 to 530 mm^2^ on day 21 (*p* < 0.0001). The slowest proliferating spheroids were U-CH12 (*p* < 0.0001), growing from 100 mm^2^ on day 4 to 180 mm^2^ on day 21 (*p* < 0.0001). This finding was confirmed via a cell survival assay. CH22, U-CH1, and U-CH12 showed an increased number of live cells during the 21 days (*p* < 0.0001, *p* < 0.01, and *p* < 0.001, respectively) with three different kinetics. Indeed, CH22 was the most proliferative (from 1.5 luminescence fluorescence intensity (LFI) on day 4 to 6.25 LFI on day 21) and reached a peak of proliferation between day 7 and day 10. U-CH1 were less proliferative than CH22 and proliferated uniformly (1.4 to 3.5 LFI). U-CH12 were the least proliferative spheroids (0.52 to 1.3 LFI) and showed decreased proliferation after day 15 (Figure 2c). Finally, Ki67+ quantification revealed 13.4%, 31.5%, and 42.1% of proliferative U-CH12, U-CH1, and CH22 cells, respectively (Figure 2d).

These patterns of proliferation are representative of the slow growth and progression of chordomas.

Since hypoxia is both a major characteristic of chordomas and a cause of radiotherapy failure, we mapped the hypoxic regions within spheroids using pimonidazole and HIF-1α staining (Figure 2e). These hypoxic regions were localized at the center of all spheroids and exhibited a nuclear HIF-1α staining (Figure 2f). Hence, we succeeded in reproducing the hypoxic status of chordoma tumors in our spheroids.

Altogether, these results indicate that our cellular models recapitulate the production of ECM, the slow growth, and the hypoxic status of chordoma, thus mimicking a radioresistant environment.

### 3.3. Chordoma Spheroids Exhibit Different Levels of Radioresistance

**Figure 3:**
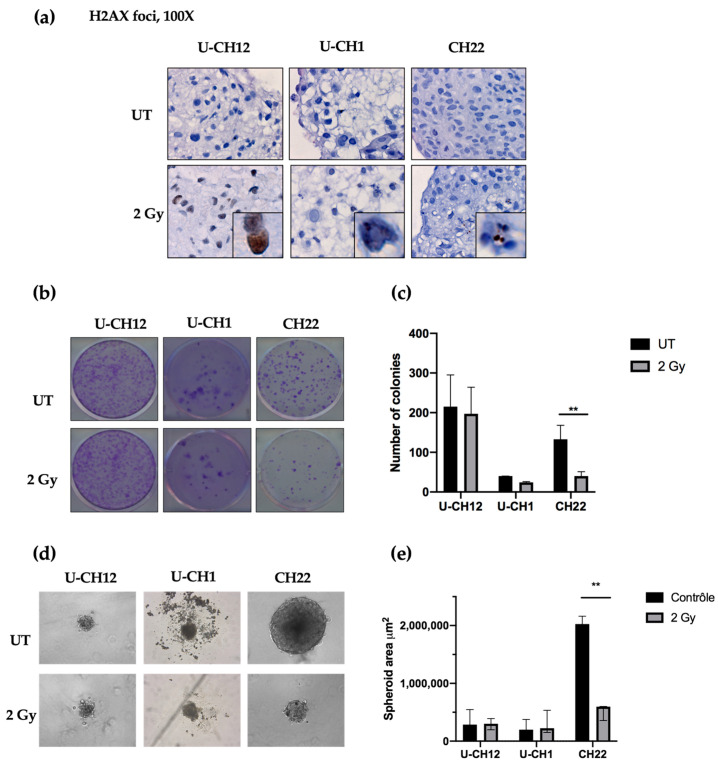
Chordomas exhibit three different levels of radioresistance: (**a**) DNA double-stranded breaks induced by radiation and quantified using γH2AX foci staining, (**b**) images and (**c**) a graph representative of the number of colonies formed in untreated conditions or after 2 Gy of X-rays, and (**d**) images and (**e**) a graph representative of the spheroid size in untreated conditions or after 2 Gy of X-rays. Each experiment was repeated three times in triplicate. Comparisons between untreated and radiation-treated groups were analyzed with a two-way ANOVA. Significant *p*-values are indicated as follows: *p* < 0.01 **.

As a control, we evaluated radiation-induced DNA damage using yH2AX-stained foci as an indicator of double-stranded breaks after radiation. Thirty minutes after being subjected to 2 Gy of X-rays, yH2AX foci were present in each spheroid, confirming the efficacy of radiation treatment (Figure 3a). Next, we examined the therapeutic effect of radiotherapy on chordoma spheroid self-renewal and proliferation. A dose of 2 Gy of X-rays resulted in a decrease in the colony forming ability of CH22 compared to that of untreated spheroids (UT: 132 colonies; 2 Gy: 40 colonies), with only 30% of colonies remaining after radiation (*p* < 0.001) (Figure 3b,c). In contrast, 2 Gy of X-rays slightly affected U-CH1 and U-CH12, with no significant difference in the number of colonies formed after treatment (UT: 40; 2 Gy: 24; UT: 215; and 2 Gy: 197, respectively) (Figure 3b,c). We then evaluated the sphere forming ability of spheroids after radiation to verify the impact on our 3D model (Figure 3d). After two weeks, the spheres newly formed from irradiated CH22 spheroids (597,609 μm^2^) had a smaller area than the spheres formed from untreated CH22 spheroids (2.10^6^ μm^2^; *p* < 0.01) (Figure 3d,e). However, the area of spheres formed from 2 Gy-treated spheroids from both U-CH1 and U-CH12 (224,048 μm^2^; 300,000 μm^2^) displayed no significant difference compared to the area of spheres formed from untreated U-CH1 and U-CH12 spheroids (198,444 μm^2^; 286,742 μm^2^) (Figure 3 d,e).

Hence, our 3 spheroids show 3 different levels of radioresistance, with U-CH12 being the most radioresistant and CH22 being the least.

### 3.4. Aldehyde Dehydrogenase Activity Is a Potential Target in Chordoma

**Figure 4:**
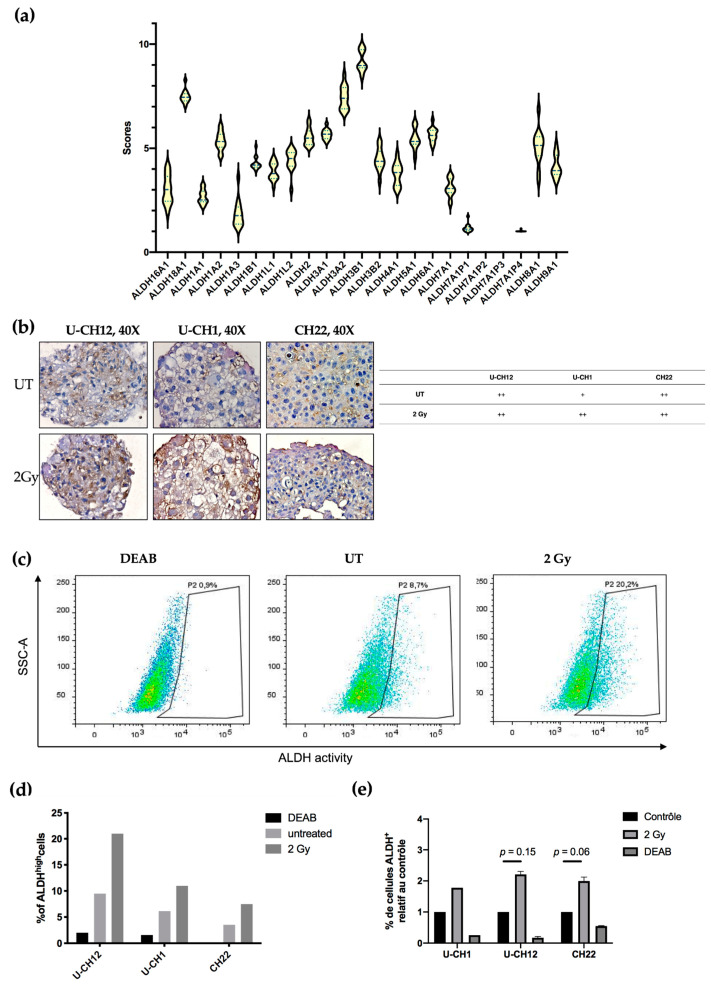
Aldehyde dehydrogenase (ALDH) is a promising radiosensitizing target in chordoma: (**a**) a graph representing ALDH gene expression scores in 13 chordoma patients, (**b**) representative images of ALDH3A2 staining after 2 Gy of X-rays or in untreated controls and quantification, (**c**) images representative of an Aldefluor assay quantifying ALDH1 and 3 activity in response to 2 Gy of X-rays in U-CH12 spheroids, (**d**) graph representative of the percent of ALDH^high^ cells, and (**e**) graph representative of the percent of ALDH^high^ cells relative to untreated conditions. Diethylaminobenzaldehyde (DEAB), an inhibitor of ALDH activity, was used as a negative control. The experiment was performed three times in triplicate for U-CH12 and CH22 spheroids, allowing for a comparison between the UT and 2 Gy groups using 2-way ANOVA. The *p*-value is indicated for each spheroid.

Aldehyde dehydrogenases are a family of detoxifying enzymes involved in radioresistance of multiple solid tumors. We initially determined whether ALDHs could be potential targets in chordoma by investigating their gene expression using RNA sequencing. A cohort of 13 patients with sacral chordoma, and both U-CH1 and CH22 spheroids were analyzed. We observed that the members of the ALDH3 and ALDH1 families were the most expressed at the transcriptomic level in all samples. Indeed, patient samples and spheroids presented high expression values of ALDH3B1, ALDH3A2, and ALDH1A2 (9, 8, and 7, respectively) (Figure 4a).

Next, we focused on the most expressed ALDH enzymes, namely ALDH1 and ALDH3. We investigated whether these enzymes are expressed in chordoma spheroids at the proteomic level. ALDH3 expression was first examined by immunostaining, which revealed that all untreated spheroids moderately expressed ALDH3. This expression increased exclusively in U-CH1 spheroids after radiation (Figure 4). We then evaluated the activity of ALDH1 and ALDH3 in spheroids by flow cytometry using an Aldefluor assay (Figure 4c–e). We used the N,N-diethylaminobenzaldehyde (DEAB) inhibitor of ALDH isoenzymes, as a negative control. These analyses showed that, at the basal level, a small subset of cells present high ALDH activity (U-CH12: 9.5%; U-CH1: 6.16%; and CH22: 3.53%). This subset of cells augments in response to radiation (U-CH12: 21%; U-CH1: 10.96%; and CH22: 7.5%) (Figure 4c), as evidenced by the 2.2-fold increase in ALDH^high^ U-CH12 cells upon irradiation (U-CH12: 2.2, *p* = 0.15; CH22: 1.9, *p* = 0.06; and U-CH1: 1.8) (Figure 4d). Interestingly, the most radioresistant spheroids, namely U-CH12 spheroids, present the most important population of cells with a high ALDH activity.

Altogether, these results show that aldehyde dehydrogenases are expressed both at the transcriptomic and the proteomic levels in chordoma patients and spheroids. Furthermore, there is an enrichment in cells with ALDH^high^ activity after radiation. Hence, ALDH1 and ALDH3 may be promising therapeutic targets in chordoma.

### 3.5. Inhibiting Aldehyde Dehydrogenase Decreases the Radioresistance of All Three Models

**Figure 5:**
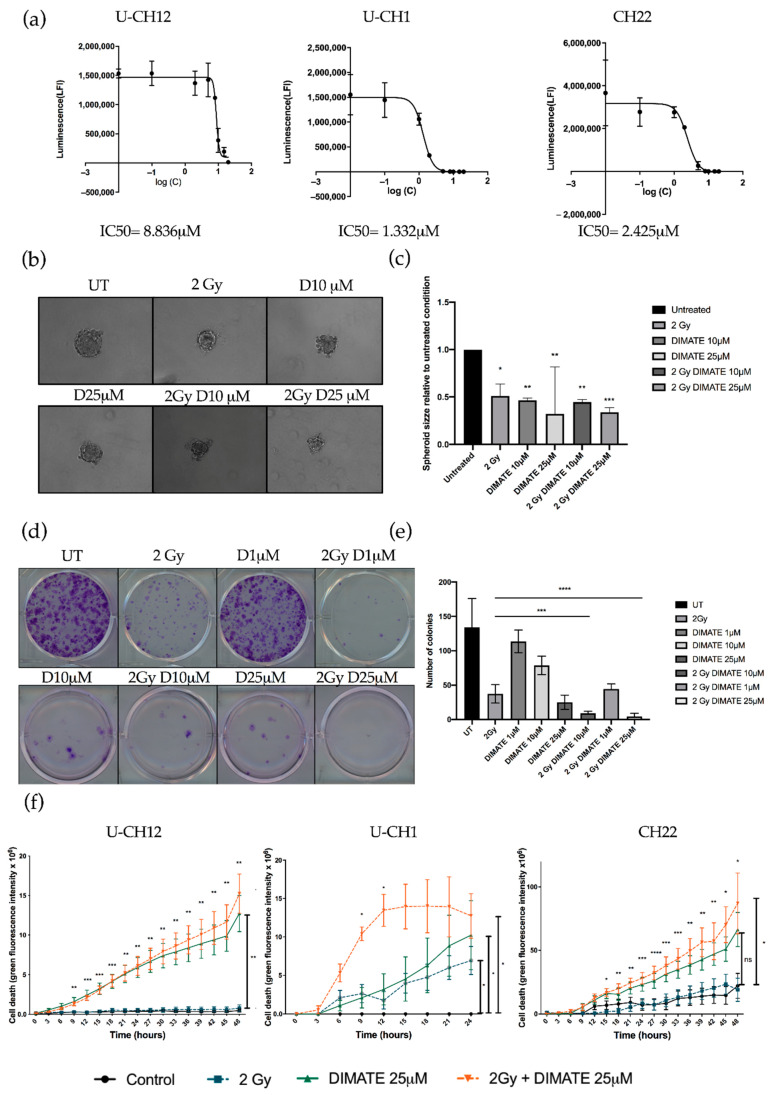
ALDH inhibition decreases radioresistance in chordoma: (**a**) a graph representative of three independent experiments of DIMATE IC50 in chordoma spheroids after 48 h of treatment at concentrations ranging from 0.1 to 25 μM, (**b**) images and (**c**) a graph representative of the CH22 spheroid size relative to untreated conditions after treatment with X-rays or after a combined treatment with X-rays and DIMATE, (**d**) images and (**e**) a graph representative of the number of colonies formed in UT conditions or after treatment with DIMATE as a monotherapy or combined with 2 Gy of X-rays in CH22 spheroids, and (**f**) a graph representative of spheroid cell death over 48 h in UT conditions or after treatment with DIMATE alone or in combination with 2 Gy of X-rays. Each experiment was conducted three times in triplicate. The statistical comparison between each group was determined using a one-way ANOVA. Significant *p*-values are indicated as follows: *p* < 0.05 *, *p* < 0.01 **, *p* < 0.001 ***, and *p* < 0.0001 ****.

We hypothesized that the dual inhibition of ALDH1 and ALDH3 could sensitize radioresistant chordoma cells. We first validated that DIMATE was able to specifically inhibit ALDH1 and ALDH3 by flow cytometry using an Aldefluor assay. Both ALDH1 and ALDH3 activity reduced strongly after DIMATE treatment (Figure S2). The IC50 of DIMATE was then established in chordoma spheroids by treating them with 0.1 to 25 μM of DIMATE. This IC50 ranged from 1332 μM in U-CH1 spheroids to 8836 μM in U-CH12 spheroids (Figure 5a). The combined treatment of DIMATE and radiations was applied to spheroids to evaluate their antiproliferative and anti-self-renewal effects. A strong decrease in colony forming ability was observed with the combination treatment at both 10 and 25 μM, with only 8 and 5 colonies remaining after treatment compared to 37 colonies remaining after radiation alone (*p* < 0.0001 for both concentrations) (Figure 5b,c). Moreover, the combination at either 1, 10, or 25 μM induced a decrease in colony forming ability more important (44, 8, and 5 colonies remaining after treatment) than DIMATE alone (113, 78, and 25 colonies remaining after treatment) at the same concentrations (*p* < 0.0001 for all concentrations) (Figure 5b,c). Hence, the combination of DIMATE and radiation triggers a stronger antiproliferative effect. This antiproliferative effect was confirmed in 3D cell cultured conditions by evaluating the capacity of cells to form spheres after the different treatment combinations (Figure 5d,e). Spheres formed from spheroids treated with the combination at 25 μM displayed a 3 times smaller area (2 Gy, D25 μM = 0.33) compared to the untreated control (UT = 1; *p* < 0.0001) (Figure 5d,e). The capacity to form spheres decreased and the newly formed spheres progressed less rapidly when spheres were formed following the combination treatment compared to those formed from radiation alone, indicative of a strong cytostatic effect.

We then evaluated whether DIMATE, radiation, or their combination induced cell death. Spheroids treated with DIMATE alone or in combination with radiation showed a strong induction of cell death after 24 or 48 h of treatment (U-CH12: *p* < 0.01; U-CH1: *p* < 0.05; and CH22: *p* < 0.05). No significant difference in the induction of cell death was observed between spheroids treated with DIMATE alone or with the combination of DIMATE and radiotherapy for U-CH12 and CH22. However, the treatment combination was more efficient at inducing cell death in U-CH1 spheroids, with a faster induction of cell death (9 h and 12 h of treatment, *p* < 0.05). Moreover, the treatment combination was more effective than radiation alone in all 3 spheroids, 9 h after treatment onwards for U-CH12 and U-CH1 spheroids (*p* < 0.01 and *p* < 0.05, respectively), and after 15 h of treatment for CH22 (*p* < 0.05) (Figure 5f). Hence, DIMATE exhibited not only cytostatic effects but also cytotoxic effects able to reinduce cell death in combination with radiation. Hence, the inhibition of ALDH1 and ALDH3 reinduced radiosensitivity in chordoma spheroids.

## 4. Discussion

Chordoma patients currently rely on aggressive surgery and high-dose radiotherapy. The infrequency of chordoma has so far delayed and obstructed the development of efficient targeted therapies against these tumors. It has been demonstrated that 95% of the drugs in phase I of clinical trials never obtain FDA approval [30]. In the last decade, efforts have been made to understand this lack of efficacy, which may partly be due to the low predictive value of preclinical models. Indeed, only a few cell lines and patient-derived xenografts (PDX) are available as in vitro and in vivo models of chordoma. To better predict response to drugs, we thus needed to create models at the crossroad between cell lines and PDXs. In this study, we established, characterized, and used 3D cellular models of chordoma for the preclinical evaluation of a radiosensitizing strategy. First, these models are representative of the histological and immunohistochemical features of the disease. Interestingly, brachyury had higher expression levels in CH22 spheroids than in U-CH12 spheroids. Indeed, brachyury overexpression has been detected in several epithelial cancers and was shown to promote the epithelial-to-mesenchymal transition that enables tumors to metastasize. The levels of brachyury expression have been correlated with disease stage, poor prognosis, and tumor resistance to cytotoxic therapies in a number of cancers [31,32,33,34]. In chordoma, brachyury overexpression is also associated with shorter progression-free survival [35]. Thus, the difference in expression of brachyury between primary and metastatic spheroids could be due to the role of brachyury in tumor progression. These models also recapitulate the peculiar radioresistant environment of chordoma and the progression of the disease (primary, relapse, and metastatic).

The current limitations of cell culture are now partially overcome by 3D cell cultures. Three-dimensional cell cultures better mimic the native cancer tissue, since they restore some of the specific biochemical and morphological features seen in vivo [36,37]. In our models, important morphological features were observed with the presence of the extracellular matrix in U-CH12 and U-CH1 composed of proteoglycans and collagens. Moreover, while cells are equally exposed to drugs in 2D cultures, in chordoma 3D models, we recapitulated the difficulty of drugs to gain access to tumor cells. Over the last few years, new complex 3D models have emerged, with the most interesting one remaining organoids. Organoids allow us to collect the primary culture of samples from patients and to maintain intra-tumoral heterogeneity [38]. One study has so far reported the establishment of organoid models of chordomas in a preclinical context to predict the response to PD-1/PD-L1 checkpoint inhibitors [39]. Despite these promising results, a deeper characterization of these primary cell cultures is needed. Chordoma spheroids contain different types of cells: proliferating, quiescent, hypoxic, and mimicking the diversity found within a tumor cell. Moreover, in vivo models of chordoma are interesting because they are able to progress. However, owing to the scarcity of chordoma samples and the slow growth rate of these tumors, organoid development has been hindered. Here, we addressed tumor progression by using cell lines established from a primary, a relapsed, and a metastatic tumor from different patients. Finally, our models match the clinical description of chordoma: primary tumors have low cellularity, slow growth, and an abundant extracellular matrix, while metastatic tumors have high cellularity, faster growth, and less extracellular matrixes.

Preclinical in vivo models are also of utmost importance to determine the bioavailability of drugs in a living organism. Giving the differences between our 3D models, it was not surprising to obtain variable levels of radioresistance, reflecting what is observed in the clinic [6]. As patients with sacral chordomas are radioresistant, they are treated with fractionated high-dose radiotherapy with a maximal dose of 2 Gy/per session [6]. We also subjected our models to this dose to mimic clinical settings. To our knowledge, only two studies have so far focused on chordoma radioresistance. The first study demonstrated that U-CH1 cells grown in 2D have a normal radiosensitivity range, whereas we found a strong radioresistance [40]. This difference may be due to an alteration in the cell line used, as the authors kept only one highly dividing subclone. We thus speculate that the quiescent and low dividing subclones must be at the origin of radioresistance in this cell line. Confirming this hypothesis, the second study evaluated the response of the U-CH1 cell line to ionizing radiation and found the same rate of survival at 2 Gy of X-rays as ours [41]. The variable degrees of radioresistance found can also be explained by the differences in proliferation, composition of extracellular matrix, and hypoxia. The extracellular matrix is known to increase cell adhesion and drives radioresistance. Moreover, hypoxia is a cause of treatment failure, and new radiotherapeutic strategies aim at delivering a stronger dose to hypoxic tumor zones to eliminate chordoma tumors [13,42]. In the same intent of improving chordoma patient response to radiotherapy, proton irradiations are used in chordomas in recent clinical trials and show better results than X-rays [43,44]. Even though proton therapy is still poorly accessible, it could be interesting to study the response of our spheroids to this kind of treatment. The chordoma models we established present 3 different levels of radioresistance; hence, such models can be very helpful to evaluate radiosensitizing therapies and to study and compare their effects on low or highly radioresistant cells.

We thus also tested a radiosensitizing approach based on ALDH targeting. Aside from their role in protection against oxidative stress as detoxifying enzymes [15], ALDHs are involved in stem cell maintenance and are the principal marker of cancer stem cells [45]. ALDH1 and ALDH3 are the most extensively studied ALDHs in cancer. Cancer stem cells have increased activity of ALDH1 and ALDH3, and this is particularly used to isolate them from tumor bulk. They are involved in resistance to both chemo- and radiotherapy in most solid tumors [16,17,18,21,46]. In chordoma, the ALDH activity has been measured in cell lines. These studies demonstrated that a population of U-CH1 cells (between 0.48 and 2.5% depending on the study) have a high ALDH activity [23,24]. In comparison, we found a more important basal rate of ALDH^high^ cells (5% in UT conditions). This can be explained by the fact that we grew the cells in 3D. Indeed, in another study, the JHC7 cell line presented an increase in the ALDH^high^ population when cultured in 3D in comparison to adherent cells cultures [25]. In those studies, the ALDH activity was used to isolate cancer stem cells, and this activity was correlated with increased tumor initiation capacities and with the expression of genes involved in stem cell maintenance. Cancer stem cells have also been detected in chordoma patients and cell lines (JHC7, U-CH1, and U-CH2) [23,24,25,47]. Here, we confirmed the presence of a subpopulation of cells with a high ALDH activity in 3 different models, but we also correlated this activity with resistance to radiation. This subpopulation of cells could potentially be stem cells.

Finally, we tested the efficacy of an inhibitor of ALDH1/3 (DIMATE). DIMATE has well-known antiproliferative effects in acute myleoid leukemia, non-small cell lung cancer (NSCLC), melanoma, and prostate cancer in vitro [26,48,49,50]. This inhibitor has been used as a monotherapy or combined with a chemotherapeutic agent inducing ROS production in NSCLC, with the aim of disrupting ROS balance in cells to induce death. The combination of DIMATE and CDDP in NSCLC has a synergistic effect on cell death in vitro and in vivo [26]. Here, we combined DIMATE with radiotherapy (X-rays). An important part of the effects produced by radiotherapy rely on ROS, which enhance DNA damage and lead to cell death. Our results show that the combination of DIMATE and radiation induces a strong inhibition of self-renewal capacities with complete inhibition of both the colony forming and the sphere forming abilities. Moreover, an important induction of cell death was observed with DIMATE both in mono- and combined therapy. The induction was accelerated in U-CH1 spheroids when the treatments were combined. Such an effect has never been obtained in vitro in chordoma, making ALDH a promising target for future preclinical experiments. Interestingly, the inhibition of RAD51 has been shown to sensitize chordoma cells to radiations in vitro [41]. Moreover, this inhibition also sensitizes cells to aldehyde treatment, substrates of ALDH activity [51]. As our work shows that the inhibition of ALDH activity sensitizes cells to radiations, we wonder whether the inhibition of both RAD51 and ALDH could have synergistic effects and improved response to radiation in chordoma. In conclusion, we created 3D cellular models of sacral chordoma derived from primary, recurrent, and metastatic tumors that reproduce histological and therapeutic features of the disease. These models can be used to decrypt chordoma alterations, in mechanisms of progression, and for preclinical evaluation of drugs. We show for the first time the benefits of using dual inhibitors of ALDH1 and 3 as radiosensitizing agents in chordoma in vitro and provide preclinical support for their use as monotherapies or in combination with radiotherapy in chordoma.

## Figures and Tables

**Table 1:** Patients clinical informations

Age	Sex	Tumor Location		Treatment	Local Relapse or Metastasis
84	F	Sacrum	Primary	Surgery	No
77	F	Sacrum	Primary	Surgery	No
70	M	Sacrum	Primary	Surgery	Local relapse
59	F	Sacrum	Primary	Surgery	Local relapse
67	M	Sacrum	Primary	No surgery	No
70	M	Sacrum	Primary	No surgery	No
56	M	Sacrum	Primary	Surgery	No
36	M	Sacrum	Primary	RT	No
66	M	Sacrum	Primary	Surgery	No
72	F	Sacrum	Primary	Surgery	No
57	M	Sacrum	Primary	Surgery	No
40	M	Crane	Metastasis	No surgery	No

## Data Availability

The data presented in this study are available on request from the corresponding author. The data are not publicly available because chordoma cohort is being expanding and more molecular analysis are ongoing and planed.

## Supplementary Materials

The following are available online at https://www.mdpi.com/2073-4409/10/2/399/s1, Figure S1: Comparison of RNA sequencing data from spheroids and chordoma patient samples; Figure S2: Graph representative of an aldefluor assay using flow cytometry after treatment with DIMATE; Table S1: Table representing the most up-regulated genes between chordoma spheroids and patient samples in the differentially expressed genes analysis; Table S2: Table representing the most down-regulated genes between chordoma spheroids and patient samples in the differentially expressed genes analysis.
