# Supplementary material for: Aldehyde Dehydrogenase, a Therapeutic Target in Chordoma: Analysis in 3D Cellular Models
Source: Cells. 2021 Feb 15;10(2):399. doi: 10.3390/cells10020399 (PMC7919493; doi:10.3390/cells10020399)
Supplement: Supplementary file 1 [file cells-10-00399-s001.pdf]

Aldehyde Dehydrogenase, a Therapeutic Target in Chordoma: Analysis in 3D Cellular Models

Supplementary Materials:

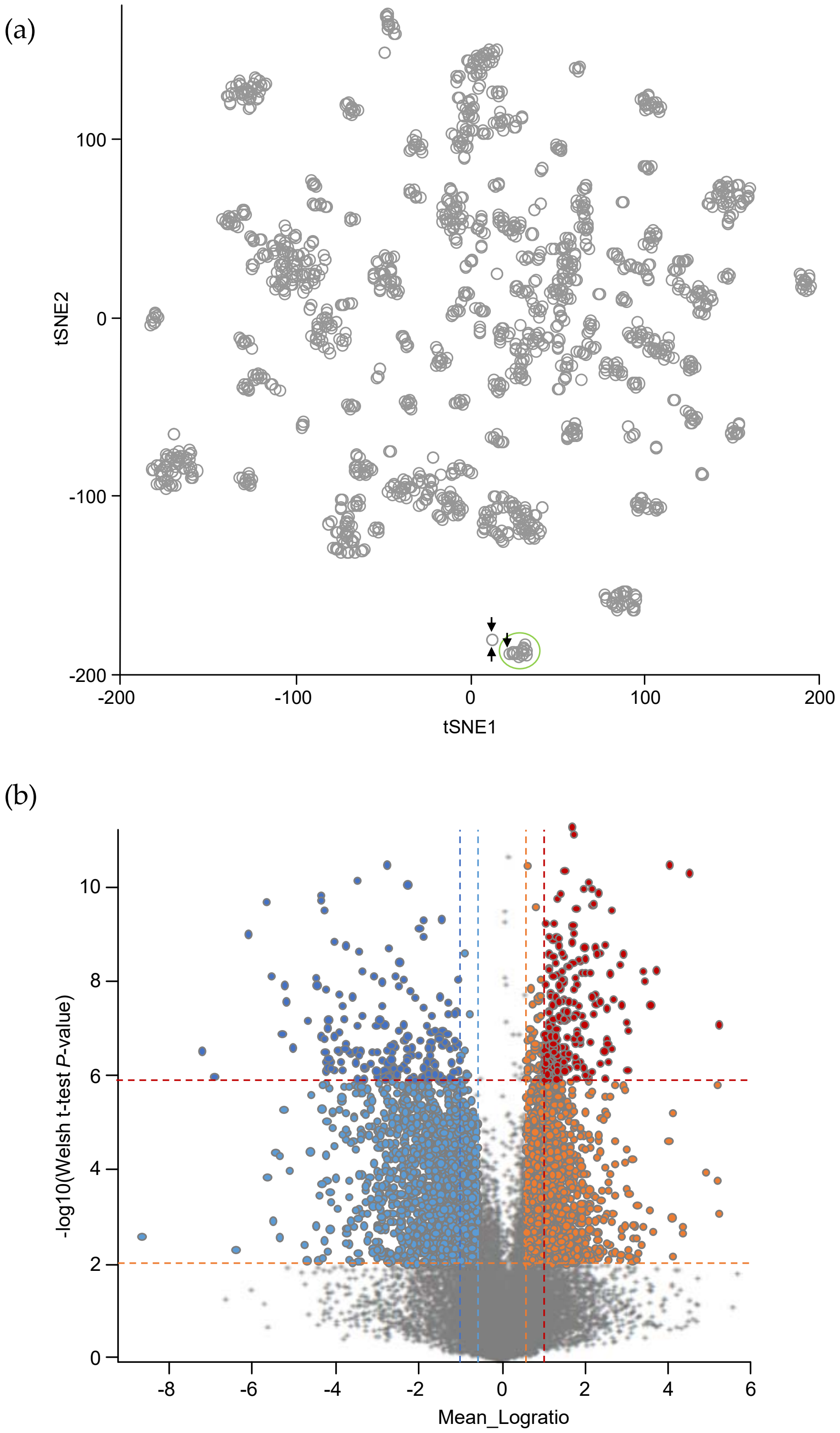

Figure S1. Comparison of RNA sequencing data from spheroids and chordoma patient samples. (**a**) tSNE visualization of 1,450 sarcoma samples including 13 chordoma samples and 3 chordoma spheroids (black arrows), with at least 144 molecular subtypes. (**b**) Volcano plot of the differentially expressed genes between chordoma samples and spheroids.

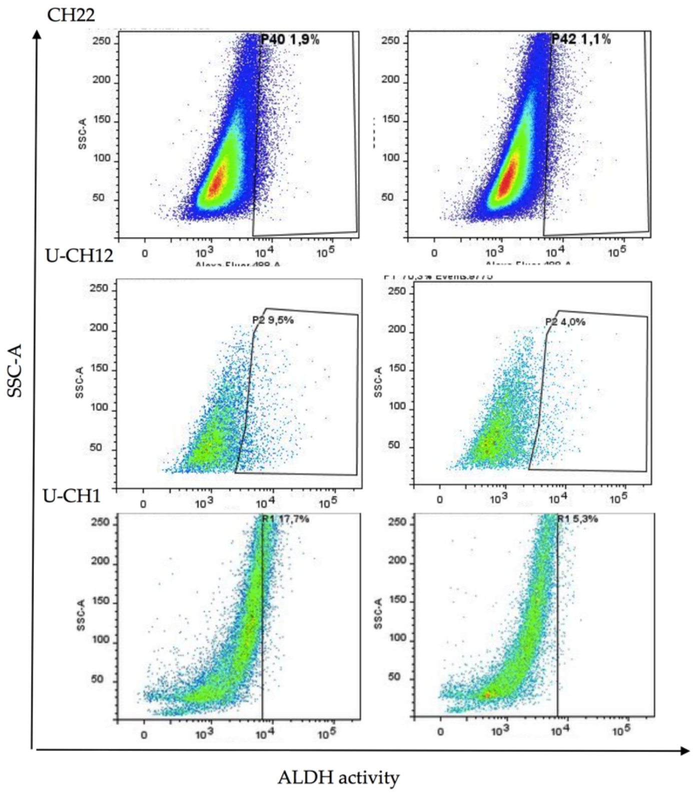

Figure S2. Graph representative of an aldefluor assay using flow cytometry after treatment with DIMATE. DIMATE induced a decrease in ALDH activity.

Table S1. Table representing the most up-regulated genes between chordoma spheroids and patient samples in the differentially expressed genes analysis.

Up-regulated Genes	Fold change	Bonferroni-corrected_P-value	Description
WDR62	5.617079927	6.64E-05	LIM homeobox 2 [Source:HGNC Symbol;Acc:HGNC:6594]
CCDC150	5.079177241	9.68E-05	bolA family member 2B [Source:HGNC Symbol;Acc:HGNC:32479]
NECAB3	4.763940807	7.54E-05	solute carrier family 47 member 1 [Source:HGNC Symbol;Acc:HGNC:25588]
MRPL12	4.648279561	8.46E-06	H4 clustered histone 12 [Source:HGNC Symbol;Acc:HGNC:4784]
BOLA2B	4.541958656	9.56E-06	nudix hydrolase 4B [Source:HGNC Symbol;Acc:HGNC:18012]
SLC47A1	3.976180569	7.25E-05	coiled-coil-helix-coiled-coil-helix domain containing 3 [Source:HGNC Symbol;Acc:HGNC:21906]
LHX2	3.230557596	6.04E-05	LIM homeobox 2 [Source:HGNC Symbol;Acc:HGNC:6594]
GRIN2B	2.727573452	9.87E-05	glutamate ionotropic receptor NMDA type subunit 2B [Source:HGNC Symbol;Acc:HGNC:4586]
CHCHD3	2.584917492	7.06E-05	transmembrane protein 161A [Source:HGNC Symbol;Acc:HGNC:26020]
TMEM161A	2.567672072	6.79E-06	raftlin, lipid raft linker 1 [Source:HGNC Symbol;Acc:HGNC:30278]

Table S2. Table representing the most down-regulated genes between chordoma spheroids and patient samples in the differentially expressed genes analysis.

Down-regulated Genes	Fold change	Bonferroni-corrected_P-value	Description
H4C12	-49.92829814	7.92E-06	H4 clustered histone 12 [Source:HGNC Symbol;Acc:HGNC:4784]
LMX1A	-20.55931439	5.65E-06	LIM homeobox transcription factor 1 alpha [Source:HGNC Symbol;Acc:HGNC:6653]
TMEM71	-16.08483601	5.58E-05	transmembrane protein 71 [Source:HGNC Symbol;Acc:HGNC:26572]
RFTN1	-13.32514016	6.78E-05	raftlin, lipid raft linker 1 [Source:HGNC Symbol;Acc:HGNC:30278]
NUDT4B	-10.78702601	9.10E-05	nudix hydrolase 4B [Source:HGNC Symbol;Acc:HGNC:18012]
NUDT4P2	-10.78702601	9.10E-05	nudix hydrolase 4 pseudogene 2 [Source:HGNC Symbol;Acc:HGNC:18013]
SYCP2	-6.534399923	7.64E-05	synaptonemal complex protein 2 [Source:HGNC Symbol;Acc:HGNC:11490]
MACF1	-4.778987384	3.44E-06	microtubule actin crosslinking factor 1 [Source:HGNC Symbol;Acc:HGNC:13664]
ARNTL	-3.913702481	3.05E-05	aryl hydrocarbon receptor nuclear translocator like [Source:HGNC Symbol;Acc:HGNC:701]
TAB2	-3.642458908	4.56E-05	TGF-beta activated kinase 1 (MAP3K7) binding protein 2 [Source:HGNC Symbol;Acc:HGNC:17075]
